# Prevalence, clinical characteristics, and associated factors of molar-incisor hypomineralisation among schoolchildren: a cross-sectional study in the Vietnamese Northern provinces

**DOI:** 10.1038/s41598-025-25960-y

**Published:** 2025-11-26

**Authors:** Phuong Huyen Nguyen, Thanh Ha Pham, Quang Binh Nguyen, Thi Hong Minh Nguyen, Thi Hanh Nguyen, Thi Bich Nguyet Vu, Thanh Huyen Nguyen, Quoc Hoan Nguyen, Truong Nhu Ngoc Vo

**Affiliations:** 1 School of Dentistry, Hanoi Medical University, Hanoi, 100000 Vietnam; 2 National Hospital of Odonto-Stomatology, Hanoi, 100000 Vietnam

**Keywords:** Children, Epidemiology, MIH, Molar hypomineralisation, Prevalence, Vietnam, Diseases, Health care, Medical research

## Abstract

This study aimed to analyse the prevalence, clinical status of Molar-incisor hypomineralisation (MIH), and associated factors in schoolchildren from Northern provinces in Vietnam. A cross-sectional study was performed in 2024 among 1834 7- to 11-year-old schoolchildren across three provinces. MIH and enamel defects were diagnosed using the European Academy of Paediatric Dentistry criteria. Clinical dental examinations were undertaken, and parental questionnaires collected relevant background information. The overall MIH prevalence was 12.7% (95% CI 11.2–14.3%). The lower left first permanent molar was the most affected tooth. For incisors, the upper right central incisor was most involved. Common clinical presentations included white or creamy demarcated opacities, lesions involving less than one-third of the tooth surface, and severe lesions. Except for age, there were no significant relationships between MIH and gender, geography, or most prenatal, perinatal, and postnatal factors. Postnatal pneumonia was significantly associated with MIH (OR = 1.49; 95% CI 1.000035–2.24). Hypomineralised second primary molars (HSPM) were observed in 7.1% of children (95% CI 5.96–8.36%) and increased the risk of MIH significantly (OR = 8.48; 95% CI 5.81–12.39). This study provided population-based data on MIH in Vietnam and contextualized it within global findings, highlighting the importance of early diagnosis and management, while acknowledging recent translational perspectives under the broader concept of molar hypomineralisation (MH).

## Introduction

Molar-incisor hypomineralisation (MIH) was the term first defined as “hypomineralisation of systemic origin, presenting as demarcated, qualitative defects of the enamel of one to four first permanent molars (FPMs) frequently associated with affected incisors”^1^. This condition was further described as a developmental, qualitative enamel defect that could lead to discoloration and even fractures of the affected enamel^2^. MIH caused a variety of concerns for patients, as reported in previous studies, such as the risk of pulp exposure due to post-eruptive enamel breakdown, hypersensitivity or pain leading to poor oral hygiene and dental caries, some negative impact on the behavioral health of children, aesthetic aspects in anterior teeth affected by discoloration, and the risk of tooth loss^3–5^. For dental practitioners, this condition may be a challenge because the treatment is complex and requires the consideration of several factors^6^.

The European Academy of Paediatric Dentistry (EAPD) is the primary international scientific body that studies MIH extensively and publishes policy documents^6^. The first consensus document was produced in 2010 after an Interim Seminar and Workshop^7^. More recently, the updated EAPD policy document was completed in 2021 and is the guideline for clinicians treating MIH^6^. According to the EAPD diagnostic criteria, MIH’s clinical signs and symptoms include white, creamy, or yellow/brownish demarcated opacities, post-eruptive enamel breakdown, sensitivity, atypical restorations, and extraction of molars due to MIH. EAPD also reinforced the severity of MIH as mild or severe. Studies also presented treatment methods for incisors and molars, in which resin infiltration was considered effective in masking color^8,9^.

In recent years, a translational science movement led by the D3 Group for Developmental Dental Defects (D3G) has introduced an updated framework that unifies MIH and related enamel hypomineralisation conditions—commonly termed “chalky teeth”—into a broader, scientifically strengthened concept known as Molar Hypomineralisation (MH)^10,11^. While the EAPD 2022 criteria remain the established clinical guideline for MIH diagnosis and management, the D3G translational paradigm is increasingly regarded as a scientific reference point in the field of developmental enamel defects and has been adopted by dental journals^12–14^. The D3G’s online database provides continuously updated global data on MH prevalence and serves as a complementary scientific resource, fostering harmonisation between clinical and translational understanding^15^. In line with the approach of Rodd (2021), this study applies the EAPD 2022 diagnostic criteria for MIH while acknowledging the emerging D3G paradigm as an advocate for future translational research on molar hypomineralisation^13^.

Literature reveals that the published data concerning MIH vary noticeably in different regions, countries, and areas. Jälevik (2010) reported in a systematic review that the prevalence of MIH ranged from 2.4 to 40.2%^16^, with Sydney (Australia) showing a prevalence as high as 44%. Zhao (2017) estimated the pooled prevalence from 70 studies, indicating that MIH prevalence ranged from 0.5 to 40.2%, with a pooled prevalence of 14.2% (95% CI 12.6–15.8%), particularly among children under 10 years old^17^. Lopes (2021) selected 116 studies from 50 countries across all five continents, estimating the prevalence of MIH among 113,089 participants to be 13.5% (95% CI 12.1–15.1%)^18^. Sluka (2024) performed a meta-analysis using data from published studies, revealing that the prevalence of MIH varied from 0.48 to 46.6%, with a pooled prevalence of 12.8% (95% CI 11.5–14.1%)^19^. Notably, the prevalence of MIH has shown an increasing trend, rising from 6% in the 2000s to 14% in the 2010s.

The evidence for the aetiology of MIH is still unclear. However, many studies have suggested that the multifactorial and systemic origin, combined with the exposure to environmental pollutants in the amelogenesis process, caused this condition^20^. Garot (2021) published a systematic review on updating the aetiological factors associated with MIH^21^. The study concluded that several factors linked with the development of MIH include prenatal, perinatal, postnatal, and even genetic factors. In particular, prematurity, cesarean delivery, and birth complications such as perinatal hypoxia increased the risk of forming MIH. In addition, measles, urinary tract infection, otitis media, gastric disorders, bronchitis, kidney diseases, pneumonia, asthma, fever, and antibiotic use are postnatal factors associated with MIH. EAPD policy document in 2021 also confirmed the multifactorial model of MIH etiology^6^. Previous research revealed that hypomineralized lesions on second primary molars (HSPM) are a predictor of MIH^22,23^.

Vietnam is located in Mainland Southeast Asia. In this country, compulsory education lasts 12 years, of which children have to spend 5 years at a primary school. After reviewing international published articles related to MIH, one study on developmental defects of enamel (DDE) in Vietnam’s dioxin- and non-dioxin-affected regions revealed that the DDE rate was 20.5%^24^. However, the results of this study are not representative of Vietnamese children because it was conducted on adults. In addition, enamel defects were diagnosed based on the 1992 FDI criteria, not according to the EAPD criteria. This FDI DDE index was inadequate and time-consuming, so the study’s MIH status could not be determined^2^. On the other hand, another recent study in a locality of Vietnam reported MIH data only in that province, with a small sample size and not generalizable to the whole population^25^. Therefore, additional targeting of Vietnamese schoolchildren is needed to collect epidemiologic data comparable to international information and fill the knowledge gaps. This study aims to analyse the prevalence and clinical state of MIH and other enamel defects using EAPD diagnostic criteria and to investigate potential aetiological factors related to this condition among schoolchildren in certain Northern provinces of Vietnam.

## Material and methods

### Ethical approval

The study protocol was approved by the Hanoi Medical University Institutional Ethical Review Board (HMUIRB 1084). All methods were performed in accordance with the relevant guidelines and regulations. The Hanoi National Hospital of Odonto-Stomatology approved the dental examination, while in compulsory schools, authorization was obtained directly from the institutional administrations. Informed consent was obtained from all parents or legal guardians of participants prior to data collection.

### Study design, population, and sample selection

This study was designed as a descriptive, cross-sectional, epidemiological, and questionnaire survey from March to October 2024. Children were voluntarily asked to join the dental examination, and their parents or guardians were informed about the purpose of this study before signing the consent form.

The inclusion criteria were as follows: (1) children aged 7 to 11 years, (2) having good physical and intellectual health, and (3) agreement and cooperation during the examination with parental consent. The exclusion criteria were the following: (1) children did not agree to participate or lacked cooperation, (2) guardians did not agree, (3) children were in the process of orthodontic treatment using fixed orthodontic appliances, (4) physical or mental development problems, or congenital malformations of the oral-facial region.

The sample size was calculated using the formula for a cross-sectional study^26^, with an estimated prevalence of 25% referencing a previous Vietnamese survey in 2020^27^, a precision of 3%, and a confidence level of 95%. The minimum sample size was 800, and the non-response rate was estimated at 10%, so we aimed to recruit at least 880 participants. We selected the sample schools in three northern Vietnam provinces via community-based oral health education programs. Students attending these elementary schools were invited to participate in this study.

### Calibration and reliability

Before data collection, training and calibration were performed by a pediatric dentist (PHN) who was specialized and experienced in diagnosing and managing MIH. The trainer used the MIH/HSPM clinical data long recording sheet published by Ghanim (2015)^28^. Training included diagnosing MIH or other enamel defects through photographs and re-scoring these lesions within 2 weeks. Cohen’s kappa scores were used to evaluate inter-rater and intra-rater reliability among examiners, with their values ranging from 0.66 to 0.88 and from 0.63 to 1.00, respectively.

### Data collection

The data collection method included a clinical oral examination and a paper-based questionnaire completed by the guardians or parents. The questionnaire was designed to investigate factors that may contribute to the presence of MIH. It consists of two major sections: The first component gathered demographic information such as age, gender, parental details, the child’s grade level, and the area where the study was conducted. The second section addressed potential factors contributing to MIH, including prenatal history (medications used during pregnancy, maternal health during pregnancy, allergies), birth history (mode of delivery, such as usual, cesarean, or preterm; maternal condition at birth; mother’s age; any abnormalities of the newborn), postnatal history (birth weight and current weight, current height, chronic diseases and overall health conditions, history of measles, chickenpox, influenza, respiratory diseases, fever, and antibiotic use)^21^. The questionnaire survey, accompanied by a consent form, was sent to parents through the school administration. The questionnaire was peer-reviewed by dentists who were not involved in the study, and its clarity was confirmed by ten parents of children with MIH who met the eligibility requirements.

**Fig. 1:**
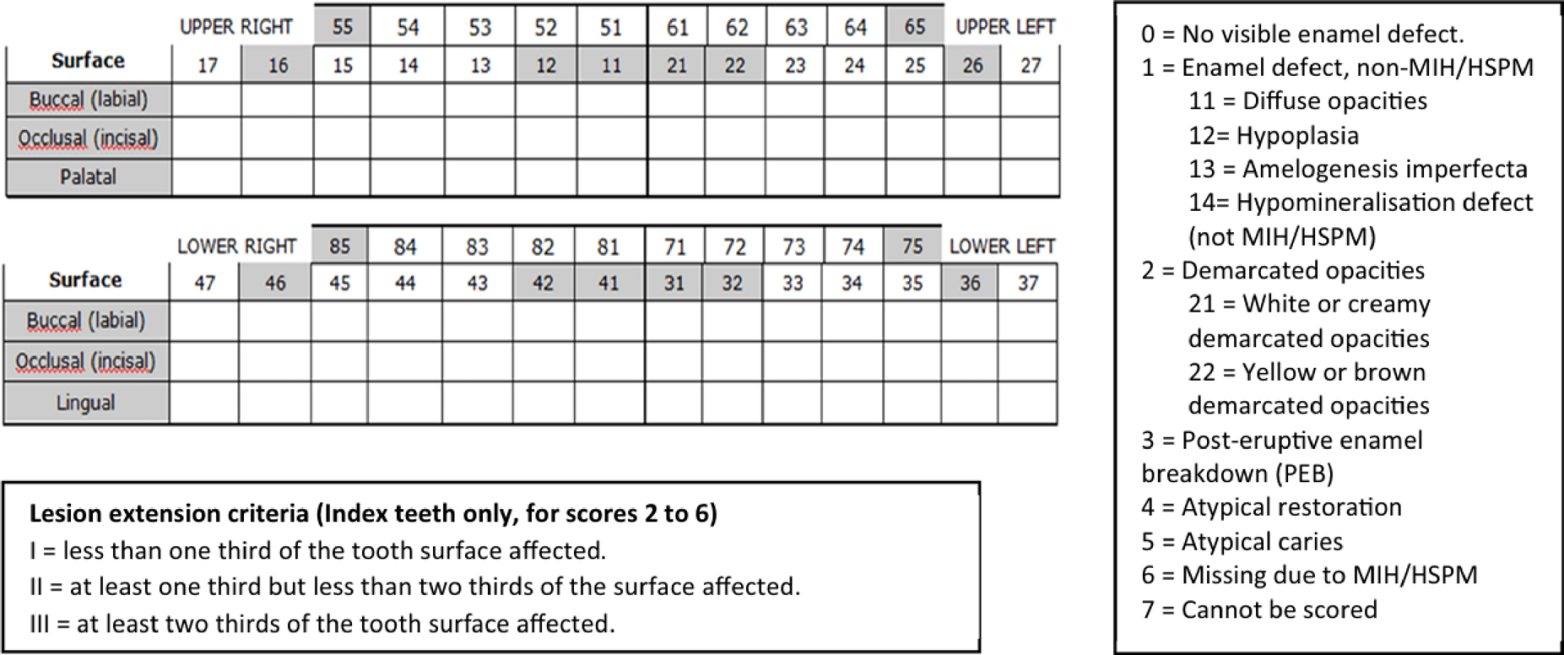
Long form for community-based study using EAPD and mDDE criteria proposed by Ghanim^4,28^.

The dental examination in children was conducted by trained and calibrated dentists according to the clinical oral health survey guidelines approved by the World Health Organization^29^. The examination used sterile mouth mirrors, gauze, dental probes, dental gloves, a forehead lamp, and Fiber-Optic Transillumination (EP light) under natural environmental light. At the same time, children were examined right at their classroom seats. The presence of MIH was recorded using the long-form sheet developed by Ghanim (Fig. 1)^4,28^. Each permanent first molar and incisor was examined on all three surfaces, occlusal, facial, and palatal or lingual, to detect enamel defects, including MIH. Teeth were numbered and named according to the World Dental Federation notation system. Each participant’s clinical status of enamel defects was scored according to the EAPD diagnostic criteria: no visible enamel defect, diffuse opacities, hypoplasia, amelogenesis imperfecta, hypomineralization defect (not MIH/HSPM) (Fig. 1). MIH was diagnosed if one or more first permanent molars were affected, with or without involvement of the incisors, including demarcated opacities, post-eruptive enamel breakdown, atypical restorations/caries, or missing teeth due to MIH/HSPM. The extent of the lesion was assessed as follows: < 1/3, between 1/3 and 2/3, and ≥ 2/3 of the tooth surface affected. The severity of MIH was categorized as mild or severe, as described in the EAPD policy document^6^. An affected tooth was considered mild MIH if it exhibited only color change or demarcated opacities. In contrast, a tooth was classified as severe MIH if the lesions involved demarcated opacities with post-eruptive enamel breakdown, atypical restorations, atypical caries, or missing teeth.

### Data analysis

Data were stored and analyzed with Microsoft Excel (Microsoft Corporation, USA) and SPSS software version 20 (IBM Corp., NY, USA). Descriptive statistics were employed to summarize the information. Quantitative variables were presented as means and standard deviations (SD), whilst categorical variables were presented as frequencies and percentages. The Chi-square and Fisher’s exact test were used to investigate proportional differences depending on the data distribution. The t-test was performed to compare mean values. Univariate logistic regression analysis was conducted to identify potential associated factors. Odds ratios (ORs) and their corresponding 95% confidence intervals (CIs) were calculated to evaluate associations between risk and protective factors within the study population and to investigate whether children with HSPM were more likely to be affected by MIH. A *p* value less than or equal to 0.05 was considered statistically significant.

## Results

During the cross-sectional study, 2429 children were invited to participate. The survey questionnaires and clinical examinations were administered. However, responses were received from 1834 children, resulting in a response rate of 75.5%. Among the respondents, 300 (16.4%) resided in the urban area of Bac Kan, 605 (33%) in Dien Bien, and 929 (50.6%) in Hai Duong. The distribution of males and females was nearly equal, with 933 males (50.9%) and 901 females (49.1%). The study sample included children aged 7 to 11 years, with 16.8% aged 7 years, 17.9% aged 8 years, 27.0% aged 9 years, 23.1% aged 10 years, and 15.2% aged 11 years, with the highest proportion in the 9-year-old group. The mean age was 9.02 ± 1.3 years.

### Distribution of enamel defects

**Fig. 2:**
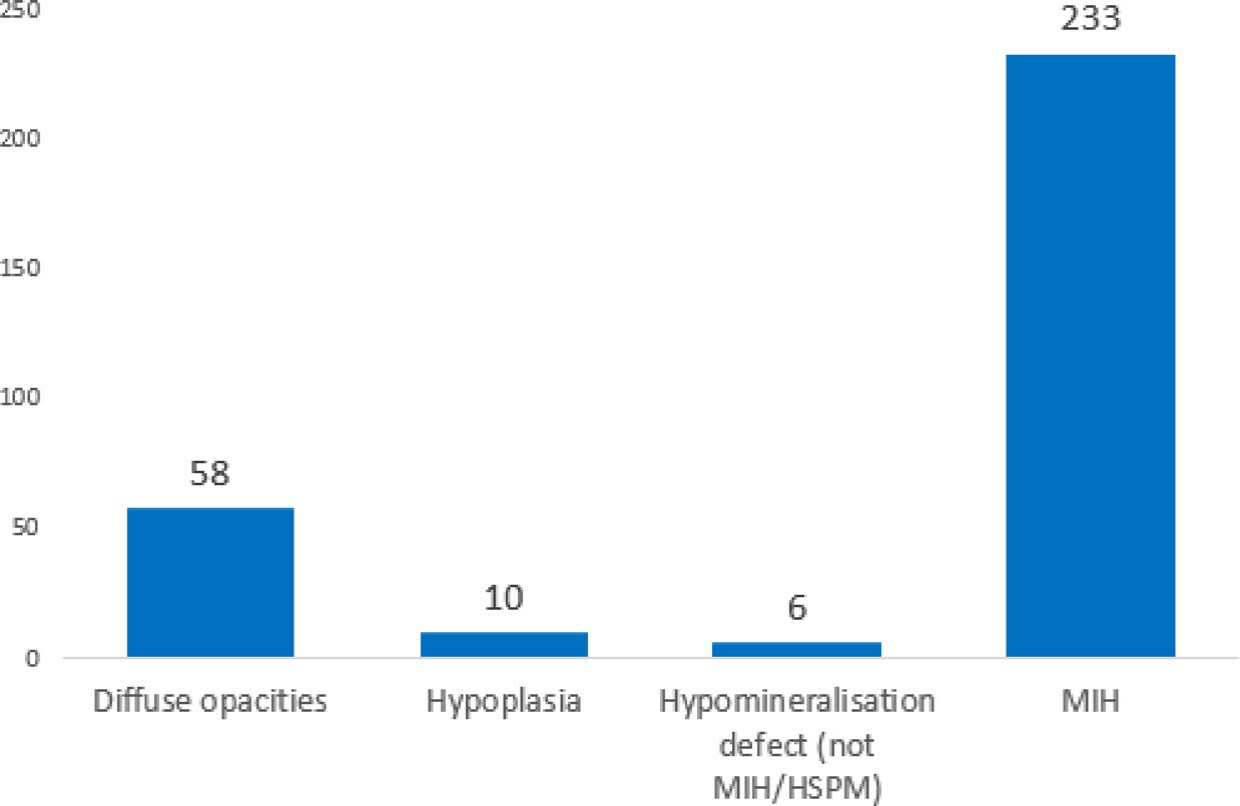
Distribution of enamel defects (N = 307).

Two hundred thirty-three children were diagnosed with MIH. Therefore, the overall prevalence of MIH was 12.7% (95% CI 11.2%–14.3%), making this condition the most prevalent. Figure 2 illustrates the prevalence of other enamel defects noted in this study. In total, 307 patients were diagnosed with enamel defects, including MIH, of which diffuse opacities accounted for 3.2% (N = 58). 0.5% (N = 10) presented hypoplasia, and the percentage of the hypomineralization defect (not MIH/HSPM) was only 0.3% (N = 6). No amelogenesis imperfecta was detected.

### Distribution of gender, age, region, and teeth affected by MIH

Of the 233 MIH children, 111 were males (47.6%), and 122 were females (52.4%). MIH was more prevalent in females than males (13.5% vs 11.9%, respectively); however, there was no statistically significant difference between the two genders (*p* = 0.291). Regarding the distribution of MIH by age, the mean age was 8.1 ± 1.4 years. It was found that a higher proportion was reported in 10-year-old children (17.7%) compared to 9-year-olds (14.1%), 11-year-olds (13.3%), 8-year-olds (8.8%), and 7-year-olds (7.1%). There was a statistically significant difference between ages regarding MIH percentage (*p* < 0.001).

Regarding the regional distribution, the MIH prevalence in Bac Kan, Dien Bien, and Hai Duong accounted for 13.7%, 12.6%, and 12.5%, respectively. There was no statistically significant relation between the MIH proportion and place of residence (*p* = 0.860). In Bac Kan and Dien Bien, there were no statistically significant differences in the prevalence of MIH between age groups (*p* > 0.05), but a significant relation in Hai Duong (*p* < 0.001).

**Fig. 3:**
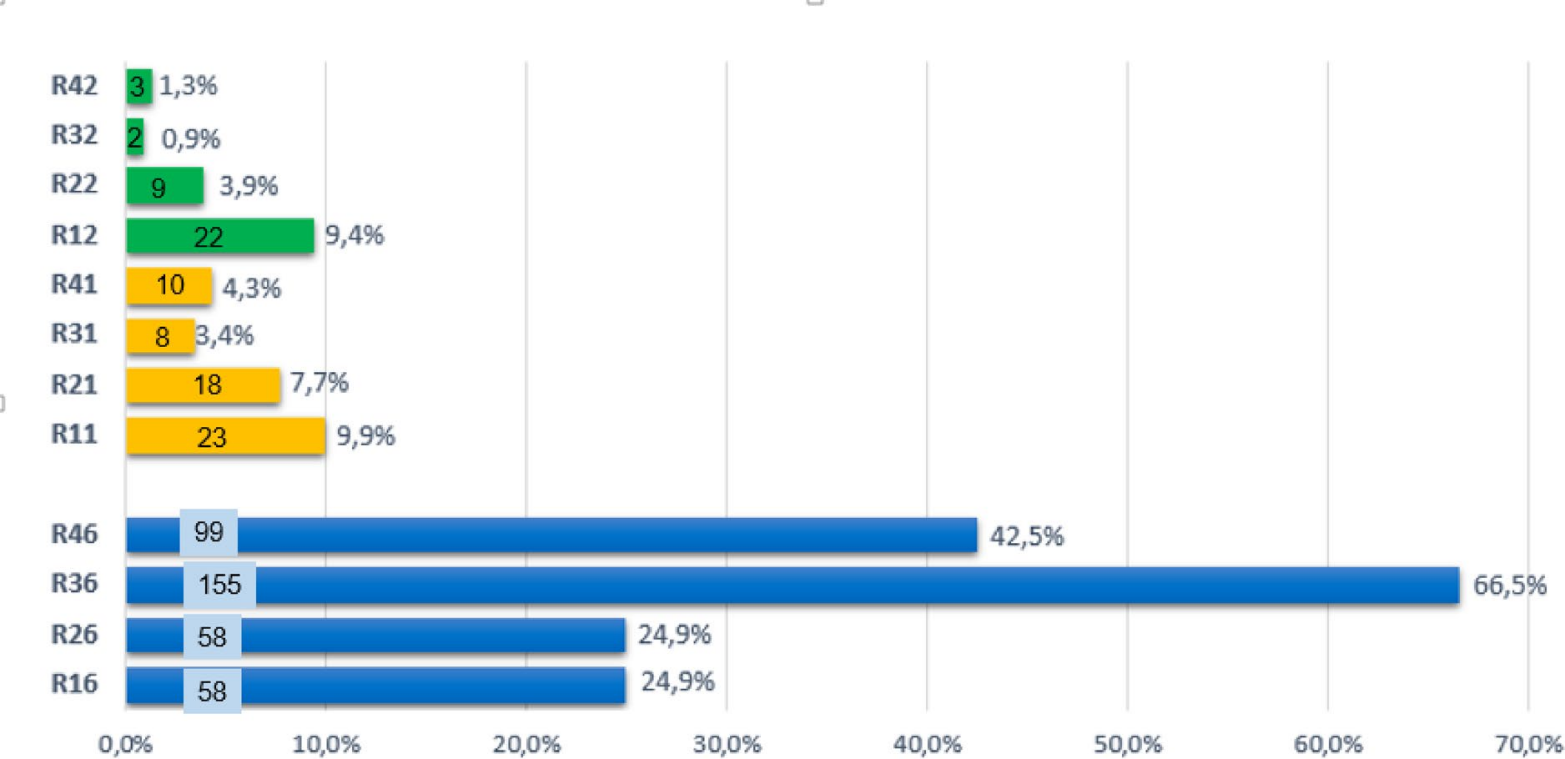
Distribution of first permanent molars and permanent incisors affected by MIH of the study population (N = 233).

In addition, the percentage of MIH lesions in each index tooth was investigated and presented in Fig. 3. There were 465 affected teeth in the 233 MIH cases, of which 370 were FPMs and 95 were permanent incisors. The lower left first molar (R36) was the most frequently affected (66.5%, n = 155), followed by the lower right first molar (R46, 42.5%, n = 99), the upper left and upper right first molar (R16 and R26, 24.9%, n = 58 for each tooth group). The most frequently affected incisor was the upper right central incisor (R11, 9.9%, n = 23), followed by the upper right lateral incisor (R12, 9.4%, n = 22), while the least was the lower left lateral incisor (R32, 0.9%, n = 2).

In the molar group, mandibular molars exhibited a higher prevalence of MIH (66.5%/42.5%) compared to maxillary molars (24.9%/24.9%), with a statistically significant difference (*p* < 0.001). Additionally, within the same group, the left side was notably more affected (24.9%/66.5%) than the right side (24.9%/42.5%) (*p* < 0.001). Regarding the incisor group, maxillary incisors (n = 72) were significantly more affected than their mandibular counterparts (n = 23) (*p* < 0.001). Furthermore, central incisors (n = 59) demonstrated a significantly higher frequency of MIH compared to lateral incisors (n = 36) (*p* = 0.015). In addition, it was found that there was a significant difference between total left (n = 37) and total right (n = 58) incisors (*p* = 0.027).

### Distribution of lesions by their extension, clinical characteristics, and severity on MIH-affected teeth

Table 1 shows MIH lesions’ distribution, extension, and clinical status according to the EAPD diagnostic criteria. The mean number of teeth affected by MIH was 1.91 (SD = 1.03), with a range from 1 to 6 teeth. Among the affected children, 50.2% (n = 117) presented with lesions limited to molars, with a mean of 1.65 (SD = 0.81) affected teeth. The remaining 49.8% (n = 116) exhibited involvement of both molars and incisors, showing a higher mean number of affected teeth [2.17 (SD = 1.14)]. Cases with only one affected PFM were more prevalent (57.9%) than those with multiple PFMs affected (42.1%).

**Table 1:** Distribution, lesion extension, and clinical characteristics of MIH according to EAPD diagnostic criteria. Shown are total numbers and percentages (in brackets) for children and teeth affected by MIH with mean and standardised deviation (SD) and min–max ranges of the number of teeth (N = number of children, n = number of teeth affected).

Variable	N (%)	Mean (SD)	Min–Max
MIH distribution			
All MIH	233 (12.7%)	1.91 (1.03)	1–6
One PFM affected	135 (57.9%)	–	–
Two PFMs affected	76 (32.6%)	–	–
Three PFMs affected	13 (5.6%)	–	–
All four PFMs were affected	9 (3.9%)	–	–
Molars Hypomineralisation	117 (50.2%)	1.65 (0.81)	1–4
Molar and incisor hypomineralisation	116 (49.8%)	2.17 (1.14)	2–6
Lesion extension			
Less than one-third of the tooth surface	232 (99.6%)	1.91 (1.03)	1–6
At least one-third but less than two-thirds of the tooth surface	132 (56.7%)	1.61 (0.77)	1–4
At least two-thirds of the tooth surface	53 (22.7%)	1.58 (0.82)	1–4
Clinical status			
White or creamy demarcated opacities	93 (40%)	1.76 (0.91)	1–4
Yellow or brown demarcated opacities	26 (11.2%)	1.62 (0.80)	1–4
Post-eruptive enamel breakdown (PEB)	15 (6.4%)	1.40 (0.83)	1–4
Atypical restoration	88 (37.8%)	1.52 (0.60)	1–4
Atypical caries	54 (23.2%)	1.70 (0.77)	1–4
Missing due to MIH/HSPM	1 (0.4%)	1	1–1

Based on the extension, hypomineralisation affecting less than one-third of the tooth surface was the most frequently observed presentation (99.6%), with an average of 1.91 (SD = 1.03) teeth affected. Cases in which one-third to two-thirds of the tooth surface was affected were less common (56.7%), with a mean of 1.61 (SD = 0.77) affected teeth. Hypomineralisation extending over more than two-thirds of the tooth surface represented the least frequent pattern, detected in 22.7% of MIH cases. Regarding clinical features, the most prevalent lesion type was demarcated opacities with a white or creamy appearance (40.0%), followed by atypical restorations (37.8%) and atypical caries (23.2%). Yellow/brown demarcated opacities and post-eruptive enamel breakdown were found in 11.2% and 6.4% of cases, respectively. Missing teeth due to MIH/HSPM were observed in only 1 case.

Regarding severity, mildly affected teeth accounted for 44.7% (n = 208), while severely affected teeth represented 55.3% (n = 257). Among FPMs, severe forms were more common than mild ones (61.6% vs. 38.4%). In contrast, within the group of incisors, mild forms were more prevalent (69.5%) compared to severe forms (30.5%).

### Association of MIH and related factors and HSPM

Logistic regression analysis indicated that age could influence MIH prevalence, showing statistically significant differences (*p* < 0.05). Compared to 7-year-olds, 9-year-olds had 2.14 times higher risk of affecting, 2.79 times for 10-year-olds, and 2.00 times for 11-year-olds. In contrast, other demographic variables, including gender, region, prenatal factors, perinatal factors, and several postnatal factors, were not significantly associated with MIH (*p* > 0.05). However, the postnatal factor pneumonia demonstrated a significant association with MIH (OR = 1.49; 95% CI 1.000035–2.24; *p* = 0.05). A more detailed overview of each factor is presented in Table 2.

**Table 2:** Association between MIH prevalence and demographic and aetiological factors of the study population. Shown are the prevalence of molar-incisor hypomineralisation (MIH) with the percentages and the numbers of children affected (in brackets), odds ratios (OR) with 95% confidence intervals in brackets (95% CI) for risk comparisons for all children, age, gender, region, and aetiological factors.

Factor	MIH	OR (95%CI)	*p* value
All children (N = 1834)	12.7% (N = 233)		
Age group			
7 years	7.1% (N = 22)	1	
8 years	8.8% (N = 29)	1.26 (0.71, 2.24)	0.438
9 years	14.1% (N = 70)	2.14 (1.29, 3.54)	0.003*
10 years	17.7% (N = 75)	2.79 (1.66, 4.61)	< 0.001*
11 years	13.3% (N = 37)	2.00 (1.15, 2.48)	0.015*
Gender			
Male (N = 933)	11.9% (N = 111)	1	
Female (N = 901)	13.5% (N = 122)	1.16 (0.88, 1.53)	0.291
Region			
Bac Kan (N = 300)	13.7% (N = 41)	1	
Dien Bien (N = 605)	12.6% (N = 76)	0.91 (0.60, 1.36)	0.641
Hai Duong (N = 929)	12.5% (N = 116)	0.90 (0.61, 1.32)	0.595
Prenatal factors			
Illness (N = 273)	15.02% (N = 41)	1.26 (0.87, 1.81)	0.214
Urinary disease (N = 42)	21.4% (N = 9)	1.91 (0.90, 4.04)	0.091
Fever (N = 57)	10.5% (N = 6)	0.80 (0.34, 1.89)	0.617
Pre-eclampsia (N = 0)	0		
Gestational diabetes (N = 15)	6.7% (N = 1)	0.49 (0.06, 3.73)	0.490
Gestational hypertension (N = 11)	9.1% (N = 1)	0.69 (0.09, 5.38)	0.720
Perinatal factors			
Child delivery			
Caesarean (N = 757)	12.6% (N = 95)	1	
Normal (N = 1077)	12.8% (N = 138)	1.02 (0.77, 1.35)	0.867
Jaundice (N = 234)	10.3% (N = 24)	0.76 (0.49, 1.19)	0.230
Low birth weight (N = 68)	13.2% (N = 9)	1.05 (0.51, 2.15)	0.893
Hypoxia at birth (N = 20)	20% (N = 4)	1.73 (0.57, 5.22)	0.330
Postnatal factors			
Fever (N = 259)	11.6% (N = 30)	0.89 (0.59, 1.33)	0.559
Varicella (N = 360)	12.5% (N = 45)	0.98 (0.69, 1.38)	0.897
Measles (N = 38)	13.2% (N = 5)	1.04 (0.40, 2.69)	0.932
Rash (N = 78)	10.3% (N = 8)	0.78 (0.37, 1.64)	0.508
Otitis media (N = 169)	13.6% (N = 23)	1.09 (0.69, 1.73)	0.711
Bronchitis (N = 295)	11.9% (N = 35)	0.91 (0.62, 1.34)	0.636
* Pneumonia* (N = 192)	17.2% (N = 33)	1.49 (1.000035, 2.24)	0.05*
Tonsillitis (N = 364)	11% (N = 40)	0.82 (0.57, 1.17)	0.273
Rhinitis (N = 202)	11% (N = 22)	0.82 (0.52, 1.31)	0.413
Asthma (N = 27)	11.1% (N = 3)	0.56 (0.26, 2.87)	0.802
Sinusitis (N = 27)	11.1% (N = 3)	0.86 (0.26, 2.87)	0.802
Throat infections (N = 15)	6.7% (N = 1)	0.49 (0.06, 3.73)	0.490
Allergies (N = 100)	8% (N = 8)	0.58 (0.28, 1.21)	0.151
Family history (N = 287)	14.29% (N = 41)	1.18 (0.82, 1.69)	0.382

*statistically significant.

Among the 1834 children assessed, 130 were diagnosed with HSPM, corresponding to a prevalence of 7.1% (95% CI 5.96–8.36%). Of the children with MIH, 27.04% (63 out of 233) also presented with HSPM. HSPM was associated with a significantly increased risk of MIH, with an odds ratio of 8.48 (95% CI 5.81, 12.39; *p* < 0.001).

## Discussion

This study is one of the first to investigate MIH prevalence, clinical characteristics (using EAPD criteria), and associated factors in Vietnam, among the few from Southeast Asia. The observed prevalence of 12.7% is comparable to the global average of 12.8% reported in a 2024 meta-analysis^19^ and the 13.1% mean from a systematic review including 99 studies across 43 countries^30^.

Numerous studies have shown that MIH prevalence varies across continents, regions, and countries, where environmental and genetic factors may influence the study population. Variability may also stem from differences in sample selection, age groups, diagnostic criteria, and examiner calibration^31^. Compared to global data, the present prevalence (12.7%) is lower than the pooled estimates reported by Zhao (14.2%)^17^ and Lopes (13.5%)^18^. The prevalence of MIH in our study aligns with the prevalence reported for Asia (13%; 95% CI 10.5–15.5)^17^, but is lower than figures from Thailand (27.7%)^32^ and Malaysia (16.9%)^33^, while similar to that of Singapore (12.5%)^34^. Within Vietnam, the current prevalence is also lower than that reported in a previous study conducted in other provinces (20.1%)^27^.

In this study, the EAPD policy document in 2022 was adopted to define MIH, guide clinical assessment and diagnosis, and develop a structured recording sheet for data collection. This ensured consistent and accurate data acquisition. The target population included school-age children aged 7 to 11 years, who had a mixed dentition and whose first permanent incisors and molars had erupted. Children in this age range are also more cooperative during dental examinations, facilitating reliable data collection and improving the accuracy of MIH prevalence estimates. This age group was consistent with those used in recent studies^31,35^ and has been recommended as the preferred population for MIH prevalence surveys in children^2^.

The recorded data on enamel defects confirmed MIH as the most prevalent lesion, followed by diffuse opacities, hypoplasia, and hypomineralisation defects (not MIH/HSPM). No cases of amelogenesis imperfecta were identified. These findings aligned with those of Arheiam (2021)^36^.

Although the prevalence of MIH was higher in girls than in boys, the difference was not statistically significant, aligning with previous systematic reviews^17,18^ and original studies^31,36,37^. Age appeared to be a potential influencing factor in our research, contrasting with previous findings reporting no significant association with age^36,37^. This discrepancy may be due to differences in study populations—our sample included 7-year-olds, while Arheiam (2021) assessed children aged 8–10 years^36^, and Grieshaber (2023) examined a broader age range of 6.5–17.9 years^37^. Additional factors such as examination variability and environmental and genetic differences may also contribute. Place of residence did not appear to influence MIH prevalence in our study, which is consistent with previous findings^36,37^. However, Amend (2020) reported a significant association between MIH and place of residence when comparing rural and urban schoolchildren^35^. As our study focused on urban schools, future research is needed to explore potential differences between rural and urban populations.

Our findings showed that the lower left first permanent molar was the most frequently affected tooth, consistent with a study from Lebanon (2019)^31^. However, another study reported that the upper right first molar was the most affected^38^. The upper right central incisor was the most commonly affected incisor, and the lower left lateral incisor the least, in line with previous studies^31,36^; however, other research identified the upper left central incisor as the most affected^38^. Within the molar group, mandibular molars were more frequently affected than maxillary molars, and teeth on the left side were more frequently affected than the right, consistent with previous studies^31,39^. In contrast, Arheiam and Cho reported opposite trends^36,40^, and Buchgraber’s study found equal involvement of upper and lower molars^41^. Among incisors, maxillary central incisors were more affected than mandibular ones, aligning with findings from Elzein^31^, Cho^40^, and Buchgraber^41^. However, the total prevalence on the right side was higher than on the left, differing from Elzein’s results^31^. These discrepancies may be attributed to variations in tooth development timing across populations and ethnicities, which influence enamel mineralization and may be affected by genetic factors^42–44^. In addition, our findings confirmed that most lesions presented as demarcated opacities involving less than one-third of the tooth surface, consistent with a previous study^31^.

Regarding associated factors, Garot (2021) concluded in a recent systematic review that peri- and postnatal conditions may increase the risk of MIH^21^. Based on the literature, these factors were investigated in our study population. Among them, only postnatal pneumonia showed a statistically significant association with MIH (OR = 1.49; 95% CI 1.000035–2.24; *p* = 0.05), consistent with the findings of Garot (2021)^21^, Silva (2016)^45^, and Bukhari (2022)^46^, who also reported pneumonia as a significant risk factor in the Middle East region. Although several pre-, peri-, and postnatal conditions were associated with higher MIH prevalence, the differences were not statistically significant.

This study reported an HSPM prevalence of 7.1% (95% CI 5.96–8.36%), which is higher than the estimate of 3.6% (95% CI 1.9–6.8%) from the meta-analysis by Lopes (2021)^18^, but aligns with McCarra’s (2022) findings of a global HSPM prevalence of 6.8% (95% CI 4.98–8.86%) across 26,805 individuals^47^. HSPM was significantly associated with an increased risk of MIH in our study (OR = 8.48; 95% CI 5.81–12.39), supporting its predictive role. This is consistent with previous studies by Estivals (OR = 6.0; 95% CI 3.7–9.7; *p* < 0.0001)^48^ and Zhang (OR = 10.90; 95% CI 4.59–25.89; *p* < 0.05)^49^.

This study standardized MIH diagnosis using the EAPD criteria, employing the long form of the recording sheet to capture defects in both primary and permanent index teeth. These findings contribute to informing policy changes in dental care provision and education for Vietnamese children, supporting improved preventive and therapeutic approaches. This study also provided data on MIH prevalence to fill the knowledge gap on disease burden in Vietnam and for future disease burden studies. A limitation of the study was the lack of data on additional factors such as caries experience (DMFT/dmft) and the estimation of treatment needs. Further research is warranted to investigate the association between MIH and dental caries and explore the population’s MIH Treatment Need Index (MIH-TNI).

In the context of rapidly evolving scientific understanding, recent translational research led by The D3 Group for Developmental Dental Defects has broadened the conceptual framework of developmental enamel defects under the term Molar Hypomineralisation (MH). This scientifically driven international initiative provides continuously updated data and offers valuable opportunities to strengthen the evidence base for enamel hypomineralisation and guide more in-depth future investigations. Further research, both globally and within Vietnam, should not only apply the EAPD 2022 clinical guideline as a clinical diagnostic reference but also consider integrating translational perspectives, such as those advanced by The D3 Group, to enhance interdisciplinary understanding and improve preventive strategies.

## Conclusion

This study provided population-based evidence on the prevalence and clinical characteristics of MIH among schoolchildren in the Northern provinces of Vietnam, thereby reinforcing the need for early diagnosis and preventive management, and integration into public health policy. The prevalence observed in Vietnam was comparable to global estimates and was significantly associated with HSPM. While the current work follows the latest EAPD 2022 clinical guideline criteria, future studies may integrate the emerging translational perspective of Molar Hypomineralization (MH) to enhance global harmonization of terminology and understanding of developmental enamel defects.

## Data Availability

The data supporting this study’s findings are available from the corresponding author upon reasonable request.
